# PIP_2_-dependent coupling is prominent in Kv7.1 due to weakened interactions between S4-S5 and S6

**DOI:** 10.1038/srep07474

**Published:** 2015-01-06

**Authors:** Marina A. Kasimova, Mark A. Zaydman, Jianmin Cui, Mounir Tarek

**Affiliations:** 1 Université de Lorraine, Theory, Modeling and Simulations, UMR 7565, Vandoeuvre-lés-Nancy, F-54506 France; 2 Lomonosov Moscow State University, Moscow, 119991, Russian Federation; 3 Department of Biomedical Engineering, Center for the Investigation of Membrane Excitability Diseases, Cardiac Bioelectricity and Arrhythmia Center, Washington University in St. Louis, St. Louis, MO 63130-4862; 4 Centre National de la Recherche Scientifique, UMR 7565, Vandoeuvre-lés-Nancy, F-54506 France

## Abstract

Among critical aspects of voltage-gated potassium (Kv) channels' functioning is the effective communication between their two composing domains, the voltage sensor (VSD) and the pore. This communication, called coupling, might be transmitted directly through interactions between these domains and, as recently proposed, indirectly through interactions with phosphatidylinositol-4,5-bisphosphate (PIP_2_), a minor lipid of the inner plasma membrane leaflet. Here, we show how the two components of coupling, mediated by protein-protein or protein-lipid interactions, both contribute in the Kv7.1 functioning. On the one hand, using molecular dynamics simulations, we identified a Kv7.1 PIP_2_ binding site that involves residues playing a key role in PIP_2_-dependent coupling. On the other hand, combined theoretical and experimental approaches have shown that the direct interaction between the segments of the VSD (S4–S5) and the pore (S6) is weakened by electrostatic repulsion. Finally, we conclude that due to weakened protein-protein interactions, the PIP_2_-dependent coupling is especially prominent in Kv7.1.

Voltage-gated potassium (Kv) channels are ubiquitous transmembrane (TM) proteins, enabling the passive flow of ions across the cell membrane upon changes in the TM voltage. They consist of four subunits, each spanning the membrane six times. Four helices, S1 to S4, compose the peripheral voltage sensor domain (VSD) that undergoes a conformational change called activation in response to membrane depolarization. S4 is the most mobile element of the VSD. It contains positively charged residues that sense TM voltage and moves in response across the membrane. S5 and S6 of the four subunits form a central pore domain (PD), which constitutes the path for permeating ions. The VSD and the PD are coupled; thus the displacement of S4 triggers the channel's gating. Several studies show that this coupling is mediated by hydrophobic interactions between the S4–S5 linker and S6^1^,^2^,^3^,^4^,^5^,^6^,^7^,^8^.

In Kv7.1 channels, in addition to protein-protein interactions, another component of coupling, protein-lipid, was recently proposed^9^. This involves phosphatidylinositol-4,5-bisphosphate (PIP_2_), a minor lipid of the inner membrane leaflet^10^. For a long time PIP_2_ was known to be important for Kv7.1 activity^11^,^12^: when this lipid is depleted from the membrane, the amplitude of the ionic current upon depolarization is dramatically reduced. Recent experiments suggest that this loss of the channel's function stems from a complete decoupling of the VSD activation from opening^9^.

Performing mutagenesis of Kv7.1, the interface between the VSD and the PD was identified as a region responsible for the PIP_2_-dependent coupling^9^. Indeed, several mutations of the S2–S3 loop, the S4–S5 linker and the S6 terminus caused a severe loss-of-function due to a decreased apparent affinity of PIP_2_ and, as a result, impaired coupling. Unexpectedly, mutagenesis of nearby residues, K183 of S2–S3 and R249 of S4–S5 caused an increase of ionic current, termed gain-of-function, due to strengthening of coupling between the voltage sensor and the pore.

To date, the molecular model describing the protein-lipid component of coupling in Kv7.1 and, moreover, its relation to the protein-protein one, remains unexplored. The interactions between the loss- or gain-of-functions residues with PIP_2_ are yet to be characterized. Whether the loss-of-function residues constitute a PIP_2_ binding site or alternatively they affect protein-lipid interactions indirectly need to be further examined. What is the gain-of-function mutagenesis effect that leads to observed increase of ionic current? We attempt here to answer these outstanding questions using combined theoretical and experimental approaches.

## Results

### PIP_2_ interacts with the VSD and the PD of Kv7.1 in a state dependent manner

**Figure 1:**
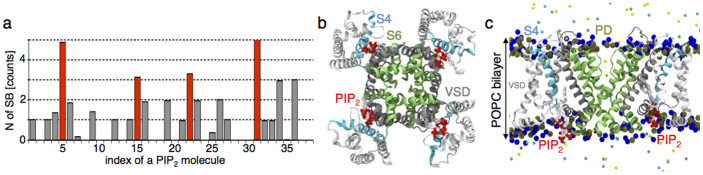
Identification of a PIP_2_ putative binding site in Kv7.1. (a) The average number of salt bridges formed between each PIP_2_ molecule (in total 38) and positive residues of the channel exposed to the interface between the lower bilayer leaflet and the solution. Red bars correspond to PIP_2_ molecules bound at the intrasubunit binding site. (b), (c) The PIP_2_ intrasubunit binding site is located at the VSD/PD interface of Kv7.1. The channel in its activated/open state is present in ribbons. Color code: the VSD and the PD are shown in white and green respectively; S4 is highlighted in cyan. PIP_2_ molecules are present as sticks with white hydrocarbon tails. Oxygen and phosphorus atoms of PIP_2_ headgroups are colored in red and tan. For clarity, only the phosphorus (tan) and nitrogen (blue) atoms of the POPC bilayer headgroups are shown. The snapshot was taken after 50 ns of the equilibration. (b) and (c) correspond to the bottom and side views respectively.

Using homology modeling^13^ and the Kv1.2 structure^14^ as a template (see Supplementary Fig. S1 for the Kv1.2/Kv7.1 alignment), we built a model of the Kv7.1 channel in its activated/open state. First, we considered the system with an excess of PIP_2_ (*cf.* Methods) and investigated the protein-lipid interactions in a well-equilibrated configuration using extensive Molecular Dynamics (MD) simulations. Within 100 ns of an unconstrained MD run, the negatively charged lipids formed either short- or long-lived (several tens of ns) salt bridges with positive residues of Kv7.1. In order to identify a PIP_2_ putative binding site, we estimated the average number of these salt bridges considering each lipid molecule individually (see Fig. 1a). Among all lipids, four PIP_2_ molecules interacted with several positive residues of Kv7.1 simultaneously, consistent with strong binding of PIP_2_ to the channel. Moreover, these molecules formed salt bridges with a terminus of S6 that composes the Kv7.1 gate (see Fig. 1a, b). Such an interaction, potentially stabilizing the positions of S6 in the open state of the channel, might hint to a reason why PIP_2_ is required for Kv7.1 to properly function. The network of salt bridges between these four lipid molecules and Kv7.1 included also residues of the S2–S3 loop and the S4–S5 linker of the same subunit. Accordingly, this PIP_2_ binding site was called *intrasubunit* (Fig. 1b). Analyzing the average number of salt bridges for prolonged trajectories (additional 20 and 40 ns), we have found similar interactions between Kv7.1 and PIP_2_ (see Supplementary Fig. S2), indicating that the performed simulations have converged within the 100 ns time scale.

Supporting our findings, the regions composing the intrasubunit site were previously identified in several experimental works as important for PIP_2_ modulation of the Kv7 family channels: S2–S3^9^,^15^, S4–S5^9^,^15^,^16^ and S6^9^,^11^,^17^,^18^. A recent molecular modeling study of Kv7.1 in the activated/open state suggested as well S2–S3 and S4–S5 as potential interaction sites of PIP_2_^15^. Specifically, the authors found that PIP_2_ interacts simultaneously with both of these regions, in agreement with our findings. However, Zhang *et al.* did not report the formation of salt bridges between PIP_2_ and the S6 terminus, which our results suggest to be crucial for PIP_2_-dependent coupling (see below).

**Figure 2:**
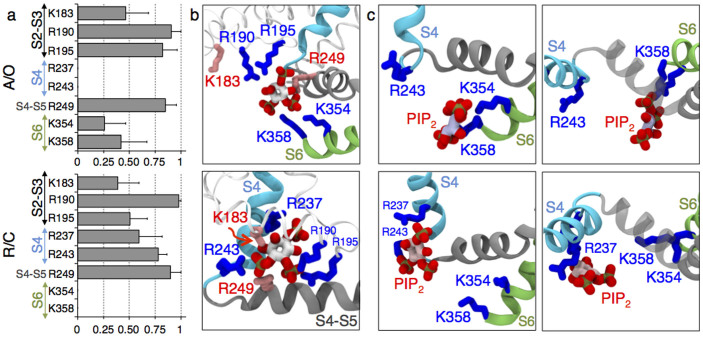
There are two modes of protein-lipid interactions each favored either in the activated/open (A/O, top panels) or resting/closed (R/C, bottom panels) states of Kv7.1. (a) Probabilities of salt bridges formation between PIP_2_ and positive residues of Kv7.1 among all subunits. Multiple MD runs are considered. The standards deviations (SDs) are represented as bars. (b) Coordination of PIP_2_ by residues composing the intrasubunit binding site: K183, R190 and R195 of the S2–S3 loop, R237 and R243 of S4, R249 of the S4–S5 linker, K354 and K358 of the S6 terminus. K183 and R249, the gain-of-function residues, are highlighted in red. (c) Side (left panel) and top (right panel) views of the representative equilibrium position of PIP_2_ at the intrasubunit binding site. Note that PIP_2_ anchors the S6 terminus in the A/O state of Kv7.1. When the channel is R/C, the lipid is shifted toward the VSD to interact with the bottom of S4.

As the overall PIP_2_ lipid content of mammalian cells' membranes is ~ 1%^10^, we tested whether the network of salt bridges was similar at low PIP_2_ concentration (*cf.* Methods). Instead of relying on one trajectory, we performed 9 independent MD runs of 100 ns each (900 ns in total) of a system containing only four PIP_2_ lipids, each initially placed at the intrasubunit binding site. 250 configurations extracted from the last 50 ns of each trajectory were considered for the probability analysis. We estimated the probabilities of salt bridges formation between specific positive residues of Kv7.1 and PIP_2_ as a ratio between the number of configurations with salt bridges formed and the total number of configurations. Of particular notice, all interactions previously identified in excess of PIP_2_ were also observed at this low concentration, suggesting that these interactions are not affected by the presence of additional PIP_2_. Specifically, R190, R195 of the S2–S3 loop and R249 of the S4–S5 linker were found to form the most frequent interactions with PIP_2_ (probability > 0.75) (Fig. 2a, b). Other residues bound to PIP_2_ with lower probabilities (between 0.25 and 0.50). These include K183 and R192 of the S2–S3 loop and importantly K354 and K358 of the S6 terminus. Finally, the residues K196 and R259 were found to interact intermittently with the PIP_2_ headgroups. The full set of interaction probabilities of all the important residues are reported in Supplementary Fig. S3. Interestingly, a single PIP_2_ molecule placed at the intrasubunit binding site formed salt bridges with both the loss- (R190, R192, R195 and K354) and the gain-of-function (K183 and R249) residues^9^.

We also investigated the resting/closed channel state. A homology model of Kv7.1 in this state was built using the structure of the resting/closed state of Kv1.2 as a template^19^,^20^. A system with four PIP_2_ molecules initially placed at the intrasubunit binding sites was considered, and 8 MD runs for 100 ns each were performed for data analyses (800 ns in total). We found that the probabilities of salt bridges formation between PIP_2_ and specific residues of Kv7.1 in this dataset were different from those in the activated/open state. Most noticeably, PIP_2_ molecules were not able to reach any of the S6 termini residues. In contrast, PIP_2_ interacted with R237 and R243, the lower positive residues of S4 (Fig. 2a, b, Supplementary Fig. S3). This interaction, never observed in the activated/open state was rather frequent (probability between 0.50 and 0.75). Finally, PIP_2_ interactions with R190, R249 and K183 remained as in the activated/open state but R195 was found to interact with PIP_2_ with a lower probability (~0.50).

Interestingly, the most probable location of PIP_2_ was slightly different in the two states of Kv7.1: the lipid was either shifted to S6 in the activated/open state or to the voltage sensor in the resting/closed state (Fig. 2c). Hence, the model emerging from the extensive MD simulations predicts two modes of protein-lipid interactions each favored in a specific channel state. These modes, hereafter called activated/open and resting/closed, differ by the residues involved in PIP_2_ coordination, and by the location of this lipid with respect to the VSD or the PD.

### Mutations of K183 and R249 gain-of-function residues favor the activated/open mode of protein-lipid interactions

**Figure 3:**
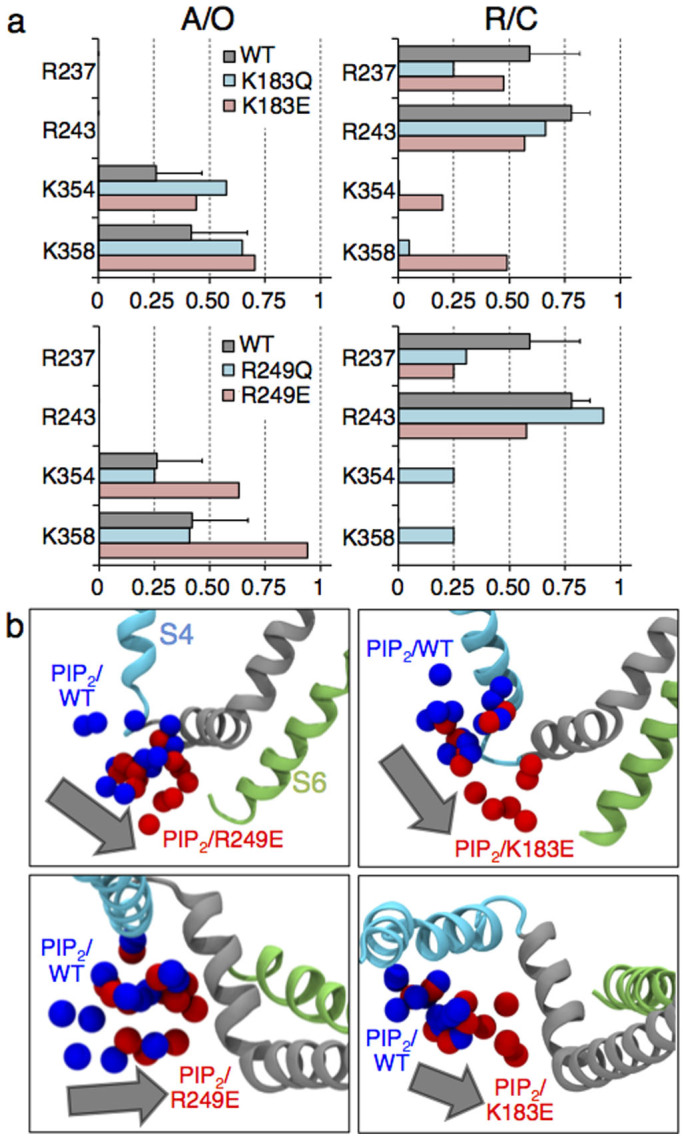
Mutations of the Kv7.1 gain-of-function residues, K183 and R249, shift the position of PIP_2_ toward S6 in both the A/O (left panel) and R/C (right panel) states of the channel. (a) Probabilities of salt bridges formation between PIP_2_ and residues of Kv7.1. (b) Shift of PIP_2_ (grey arrow) between positions in the WT (blue spheres, representing the P1, P4 and P5 atoms of the four PIP_2_ molecules) and in the gain-of-function mutants (red spheres). Here, the systems with the most significant shifts are reported: R249E in the A/O state (left panel: side and top views) and K183E in the R/C state (right panel: side and top views).

Mutagenesis of the Kv7.1 gain-of-function residues, namely K183 (S2–S3) and R249 (S4–S5), increases the ionic current^9^. In order to determine a molecular-level rational for this effect, we performed additional MD simulations of K183Q/E and R249Q/E mutants in the two states of the channel. In the resting/closed state, three mutants (K183Q/E and R249Q) had a non-zero probability to form salt bridges between S6 and PIP_2_, in contrast to the WT case, where S6 was not accessible to these lipids (Fig. 3a, Supplementary Fig. S4). In the activated/open state, the residues of the S6 helix of three mutants (K183Q/E and R249E) had higher probabilities to interact with PIP_2_ compared to the WT (Fig. 3a, Supplementary Fig. S4). Hence, going from the WT to the K183Q mutant, these probabilities increased from 0.26 to 0.60 for K354, and from 0.42 to 0.65 for K358; going from the WT to the K183E mutant, these probabilities increased from 0.42 to 0.70 for K358. Similarly, going from the WT to the R249E mutant, these probabilities increased from 0.26 to 0.63 for K354, and from 0.42 to 0.95 for K358. Note here that for each mutant in the two states only one MD trajectory of 100 ns was considered. However, the effect of mutagenesis is unambiguous as the estimated probabilities changes upon a mutation are larger than the error bars obtained for the WT. The simulations indicated also that all the mutations induced a change in the equilibrium position of PIP_2_ at the intrasubunit binding site. Indeed, in the WT resting/closed state, PIP_2_ is close to the bottom positive residues of S4, while in all four mutants PIP_2_ moved toward S6. Similarly, in the open/activated state of the mutants, the lipid was located closer to the S6 terminus and further away from the VSD compared to the WT (Fig. 3b).

In summary, neutralizing or charge reversing the gain-of-function residues, R249 or K183, induces (i) a redistribution of salt bridges between Kv7.1 and PIP_2_; and (ii) a shift of this lipid toward S6, favoring the activated/open mode of protein-lipid interactions, which is consistent with a gain-of-function effect. This result additionally validates the molecular models used here.

### The S4-S5/S6 interactions are destabilized by repulsion between their positively charged residues

**Figure 4:**
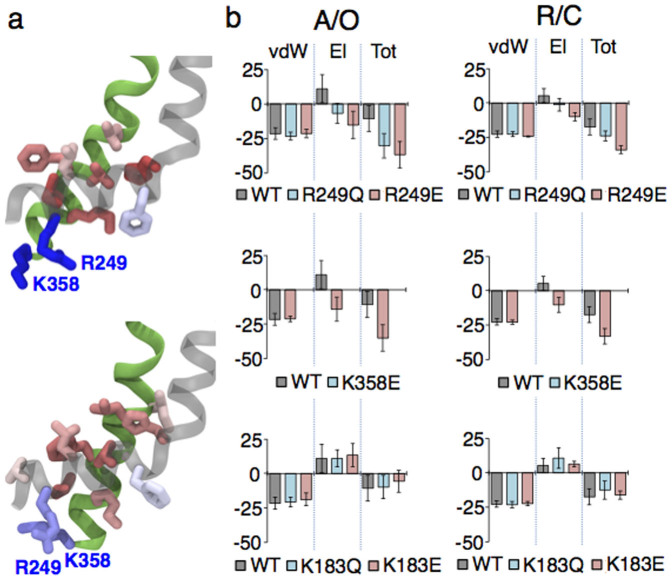
Several positive residues of S4–S5 and S6 modulate the strength of the S4–S5/S6 interactions. (a) Per-residue decomposition analysis of the nonbonded energy of interaction between S4–S5 and S6 performed in the A/O (top) and R/C (bottom) states of the WT channel. The residues are colored corresponding to their contribution. R249 and K358 shown in blue provide a positive (repulsive) contribution. Several residues highlighted in red stabilize the interaction between these two regions. (b) The nonbonded energies of the S4–S5/S6 interactions of R249Q/E (top panel), K358E (middle panel) and K183Q/E (bottom panel) compared to the WT's one reported for the A/O (left) and R/C (right) states of Kv7.1. Here vdW, El and Tot correspond to the van-der-Waals, electrostatic and total nonbonded energy respectively. The error bars (SE) are present.

We analyzed the interactions between S4–S5 and S6 in the WT channel by performing a per-residue decomposition analysis of the nonbonded energy (*cf.* Methods). The analysis revealed that, in the Kv7.1 activated/open and resting/closed states, unlike other residues, R249 of S4–S5 and K358 of S6 have a positive contribution, *i.e.* they destabilize the interaction between the S4–S5 linker and S6 (Fig. 4a). Therefore, we speculate that R249 and K358 weaken the protein-protein component of the VSD/PD coupling in the native channel.

We analyzed the nonbonded energy of the S4–S5/S6 interactions when K358 and R249 residues were mutated, considering the previously obtained trajectories for R249Q/E and a new MD run for K358E. Neutralization and charge reversal of R249 or charge reversal of K358 lowered this energy due to elimination of the electrostatic repulsion (Fig. 4b). Moreover, in both states of the channel, we observed a low probability of salt bridge formation between R249E and K358 (or R249 and K358E), which contributed additionally to the strengthening of the S4–S5/S6 interactions.

**Figure 5:**
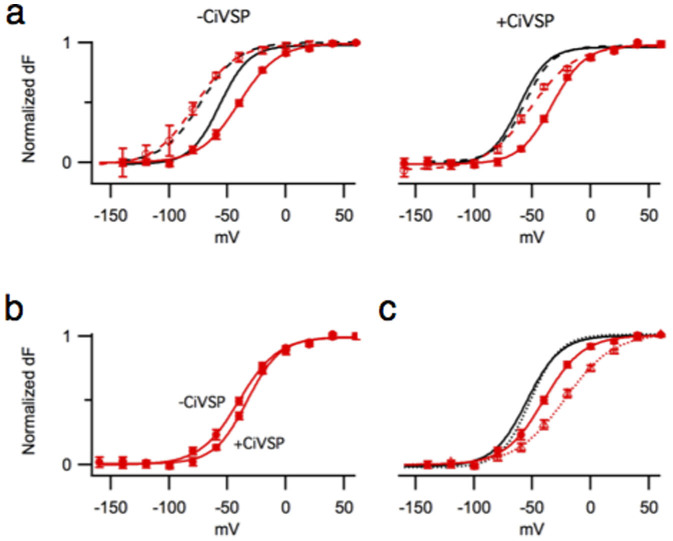
The protein-protein and protein-lipid components of coupling detected in K358E. (a) Normalized F/V curves of K358E (red solid) and K358E/L353K (red dashed) expressed alone (left) or with CiVSP (right). Normalized F/V curves of the WT (black solid) and L353K (black dashed) with and without CiVSP are shown for comparison. (b) Normalized F/V curves of K358E expressed alone or with CiVSP. (c) Normalized F/V (solid) and G/V (dashed) curves of K358E (red) and the WT (black). Note that the latter are superimposed. The WT data reproduced from^9^.

Since the molecular models suggest that the S4–S5/S6 interactions of the K358E mutant are strengthened, we attempted to detect its protein-protein component of coupling performing voltage-clamp fluorometry experiments. In our previous studies^9^, probing the Kv7.1 VSD/PD coupling we introduced the L353K mutation, which locked the channel's pore in an open state. L353 is located near the putative bundle crossing. Introduction of a positive or negative residue at this position results in a channel that remains open^21^ as if the mutual electrostatic repulsion among the introduced charges destabilizes the closed state of the pore domain. When the two domains of the L353K mutant, the VSD and the PD are coupled, the pore being locked open facilitates the activation of the voltage sensor, which can be detected as a leftward shift of the F/V curve. After PIP_2_ depletion with the voltage-dependent phosphatase CiVSP^22^ this shift is completely eliminated, indicating a predominance of the protein-lipid component of coupling in the L353K mutant^9^. However, when similar experiments were performed for the K358E/L353K double mutant, depletion of PIP_2_ reduced but did not eliminate the leftward shift of the F/V curve (Fig. 5a). Furthermore, in the absence of the lock open mutation, PIP_2_ depletion resulted in a right shift of the K358E F/V curve, as if the closed pore hindered the voltage sensor activation (Fig. 5b). This right shift was not observed in similar experiments with the WT Kv7.1^9^.

Furthermore, the F/V and G/V curves measured for the WT Kv7.1 are superimposable as if the multiple voltage sensor movements are not required for the gate opening^23^, which is consistent with rather weak coupling between the voltage sensor and the pore. However, in the K358E mutant, the F/V and G/V curves were separated by a shift (Fig. 5c), which can be interpreted as a stronger interaction between the voltage sensor and the pore in the resting/closed state compared to the WT Kv7.1. Altogether, these experiments demonstrated the presence of a lipid-independent component of coupling in K358E, which we attribute to protein-protein interactions.

**Figure 6:**
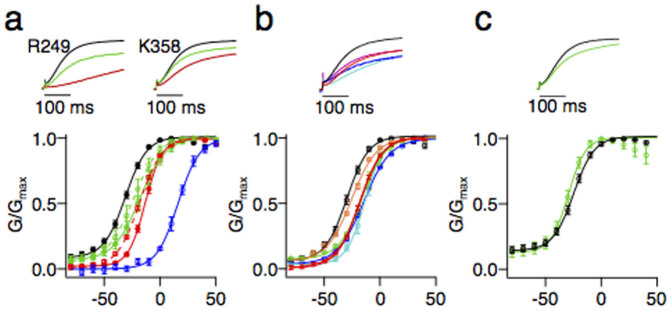
Mutagenesis of basic residues of the S4–S5 linker and the S6 terminus indicated the importance of electrostatic interactions between these two regions. (a) Normalized currents of the WT (black), charge neutralizing (green – R249Q, K358N) and charge reversing (red – R249E, K358E) mutations (top panel). The G/V curves of the WT (black), R249Q (green solid), R249E (red solid, ΔΔG = 0.56 ± 0.14 kcal/mol), K358N (green dashed), K358E (red dashed, ΔΔG = 0.68 ± 0.07 kcal/mol) and R249E/K358E (blue solid, ΔΔG = 2.60 ± 0.13 kcal/mol) (bottom panel). (b) Normalized currents (top panel) and the G/V curves (bottom panel) of the WT (black), K354E (blue), K358E (red), R360E (cyan), K362E (magenta) and R366E (orange). (c) Normalized currents (top panel) and the G/V curves (bottom panel) of the WT (black), K183N (green).

Motivated by our modeling results, we tested the importance of electrostatic interactions between R249 and K358 for the Kv7.1 gating. We found that both the R249E and K358E single mutations resulted in a delay of the current onset (Fig. 6a, top) and in a right shift of the G/V curves (Fig. 6a, bottom), consistent with a stronger interaction between the voltage sensor and the pore in the resting/closed state compared to the WT Kv7.1. In a mutant cycle analysis^24^, the double mutation R249E/K358E caused a non-additive effect on the apparent free energy of the closed to open transition (Fig. 6a, bottom). These experiments support the idea that residues 249 and 358 may interact in the WT Kv7.1. Neutralization of R249 or K358 produced intermediate effects compared to those of the charge reversal, consistent with the importance of electrostatic interactions between these two residues (Fig. 6a).

The electrostatic repulsion between the S4–S5 linker and S6 may not be limited to R249-K358 interactions. To test this hypothesis, we reversed the charges of nearby basic residues of S6. Similarly to R249E and K358E, we found that K354E, R360E, K362E, and R366E all caused a delay of the current onset (Fig. 6b, top) and a rightward shift of the G/V curve (Fig. 6b, bottom), providing evidence for the existence of a functionally significant network of electrostatic interactions between S4–S5 and S6 residues. In summary, in the WT Kv7.1, repulsion between positively charged residues on S4–S5 and S6 weakens the protein-protein component of coupling.

For completeness, we checked whether the other gain-of-function residue, K183, affected the S4–S5/S6 interactions. The analysis of the nonbonded energy for the K183Q and K183E mutants did not reveal any significant changes compared to the WT (Fig. 4b), suggesting that K183 does not affect the protein-protein component of coupling. Experimentally, unlike the R249 and K358 mutations, K183N did not cause any delay in current onset (Fig. 6c, top) or right shift of the G/V curve (Fig. 6c, bottom), confirming the model prediction that this residue is not involved in the protein-protein component of coupling.

## Discussion

Coupling is an effective communication between the VSD and the PD of a channel that provides triggering conformational changes in the pore as a response of those in the voltage sensor. Recent experiments^9^ have demonstrated that, in Kv7.1, coupling requires presence of PIP_2_. In this study, we took advantage of molecular modeling in order to uncover the details of PIP_2_-dependent coupling. The MD simulations of Kv7.1 embedded in the bilayer containing PIP_2_ revealed two modes of specific protein-lipid interactions. When the VSD is in the resting state, PIP_2_ is located close to S4 interacting with its lower residues (Fig. 2). Being close to the VSD but far from the PD, this lipid is unable to reach S6 and to hold it close to the membrane/solution interface. As a result, the pore might relax to a conformation corresponding to the closed state, *i.e.* favors the resting/closed state of the channel. In contrast, when the pore is open, PIP_2_ is shifted close to the PD and forms salt bridges with its S6 terminus (Fig. 2). Located further from S4, this lipid cannot interact with its positive residues, which potentially facilitates the activation of the voltage sensor, *i.e.* favors the activated/open state. Overall the present molecular model is in agreement with the hypothesis that PIP_2_ acts as a coupling element^9^: PIP_2_ potentially stabilizes the resting/closed and activated/open states of Kv7.1 over the resting/open and activated/closed ones.

Besides PIP_2_-dependent coupling, in Kv7.1, there are protein-protein (S4–S5/S6) interactions that mediate communication between the VSD and the PD. Here we demonstrated that these interactions are weakened by the electrostatic repulsion among positive residues of S4–S5 and S6.

A crosstalk between the protein-protein and protein-lipid components of coupling may be mediated by positive residues that are involved in the S4–S5/S6 interactions and, at the same time, coordinate PIP_2_ at the intrasubunit binding site. In the K354E and R360E mutants, for example, the protein-lipid component of coupling is potentially impaired. We assume this based on our previous findings^9^ where neutralization of these residues resulted in a severe loss of current attributed to disrupted interactions between Kv7.1 and PIP_2_. On the other hand, introducing K354E or R360E mutations provides a negative charge on S6 and should strengthen the protein-protein component of coupling, similar to the case of K358E reported above (Fig. 4, 5). Thus, despite the impaired protein-lipid component of coupling the communication of the VSD with the PD might be still ensured through the strengthened protein-protein one. Indeed, for K354E and R360E, we observed only a mild decrease of current amplitude compared to the WT (Supplementary Fig. S5), suggesting that the overall coupling between the VSD and the PD, in these mutants, is impaired only slightly.

In the MD simulations we have considered the truncated Kv7.1 channel (residues 122 to 358), neglecting therefore its cytoplasmic domain. So far, two hypothetical PIP_2_ binding sites located at this domain were proposed for members of the Kv7 family. The first one, the helix A-B linker suggested for Kv7.2-5^25^, is not relevant here since this helix linker is not conserved in Kv7.1. The second one, located at the distal C-terminus of Kv7.1, involves R539 and R555. R539W and R555C mutations (associated with long QT syndrome) were shown to decrease the channel's apparent affinity to PIP_2_^16^. Considering this effect, two hypotheses may be put forward: R539 and R555 residues either (i) form an independent PIP_2_ binding site, or (ii) contribute directly/allosterically to the intrasubunit one found here. We found that the R555C mutation eliminated the shift of the F/V curve caused by the lock open mutation, L353K (Supplementary Fig. S6a), indicating that R555C disrupts the VSD/PD coupling. Since the depletion of PIP_2_ from the intrasubunit binding site also disrupts the coupling in Kv7.1, we conclude that the distal C-terminus and the intrasubunit sites are not independent, and they affect each other either directly or allosterically. R539W had a less severe impact on the F/V curve shift (Supplementary Fig. S6b). Recent experimental findings suggest that this residue may interact with other membrane lipids besides PIP_2_^26^.

In our previous work, we have indicated importance of R259 for sensitivity of Kv7.1 to PIP_2_ lipids. In the present simulations, however, this residue interacted rather rarely with PIP_2_ placed at the intrasubunit binding site. R259 is located at the C-terminus of the S4-S5 linker and, in our molecular model of Kv7.1, is separated from the intrasubunit binding site by more than 15 Å distance and, additionally, by bulky hydrophobic F256. Interestingly, in the system with an excess of PIP_2_, we observed that four lipid molecules, distinct from those bound at the intrasubunit site, interacted with R259, which allows us to hypothesize an existence of a second binding site in Kv7.1. Indeed, recent experimental and computational studies of the Kv7.1/KCNE1 complex indicate that the TM part of Kv7.1 might comprise two PIP_2_ binding sites^27^.

The intrasubunit binding site shown here to play a crucial role in Kv7.1 is similar to the one previously found for the Kv1.2 channel^28^,^29^,^30^. In the Kv1.2 resting/closed state, PIP_2_ interacts with the bottom of S4, in particular with the lower gating charges R303, K306 and R309. In its activated/open state, PIP_2_ is shifted toward the gate anchoring R419 of S6. Hence, in Kv1.2 as well, PIP_2_ appears to interact with the VSD/PD in a state dependent manner. Despite a similar overall architecture and, moreover, a state-dependent manner of protein-lipid interactions, the response of these channels to PIP_2_ is not identical. For both Kv1.2 and Kv7.1, application of this lipid leads to an increase of ionic current^11^,^12^,^28^,^29^. However, while in the case of Kv7.1, PIP_2_ is absolutely required for opening, Kv1.2 is still able to open when this lipid is depleted. Considering the alignment between these two channels (Supplementary Fig. S1), we note that several S6 residues of Kv1.2 are not conserved in Kv7.1: (i) F416, involved in the S4–S5/S6 interactions^3^,^4^,^6^,^31^, is substituted into K354; (ii) E420, which forms a salt bridge with the S4–S5 linker (Supplementary Fig. S7), strengthening the protein-protein component of coupling, is substituted into K358. It is interesting to note that, in Kv7.1, both K354 and K358, on the one hand, destabilize the S4–S5/S6 interactions (Fig. 4a) and, on the other hand, participate in PIP_2_ coordination (Fig. 2). Furthermore, in Kv7.1, there is a long S2–S3 loop that provides two additional positive residues (R190 and R195) for PIP_2_ coordination. In Kv1.2, S2–S3 is not conserved: it is shorter by nine residues, and R195 is substituted to a neutral residue (T252). Recent modeling of Kv1.2 did not report interaction between S2–S3 and PIP_2_ in this channel^28^,^29^,^30^. These outlined differences could explain the relative predominance of the protein-lipid component of coupling over the protein-protein one in Kv7.1 compared to Kv1.2.

As Kv7.1, other members of the Kv7 family require PIP_2_ to open^11^. However, several measured characteristics of their gating are different: (i) the F/V and G/V curves of Kv7.1 overlap^23^, while the Q/V and G/V curves of Kv7.4 are separated by a shift^32^; (ii) following depolarization, in Kv7.1, current onset occurs immediately, while there is delay of current, in Kv7.2/Kv7.3 (Supplementary Fig. S8a, b); and (iii) an instantaneous current specific for Kv7.1^33^ is not observed for Kv7.2/Kv7.3 (Supplementary Fig. S8c). Looking more carefully at the channels' sequences (Supplementary Fig. S1), we noticed that several residues of S6 composing the intrasubunit binding site or located in its proximity are substituted in Kv7.2-5. Thus, K358 is neutralized (Q), and two nearby residues, Q357 and N365, are substituted to a glutamate. When we reversed the charge of K358, in Kv7.1, we observed similar gating features as Kv7.2-5 ones: (i) the F/V and G/V curves were separated (Fig. 5b); (ii) current onset was delayed (Fig. 6a, top); and (iii) the instantaneous current was reduced (Fig. 6a, bottom). Furthermore, other charge reversal mutations of S4–S5 and S6 also resulted in delayed onset and reduced instantaneous current (Fig. 6b). These results suggest that different gating among the Kv7 family members might be attributed to different strength of their S4–S5/S6 interactions.

Alternatively, different gating properties of Kv7.2-5 compared to Kv7.1 might be due to the change of protein-lipid interactions, *i.e.* the presence of other PIP_2_ binding site(s) in these channels. Indeed, a difference in protein-lipid interactions between Kv7.1 and Kv7.2 was recently highlighted by Zhang *et al.*^15^. Additionally, while in Kv7.2-5 the helix A-B linker is suggested to compose another PIP_2_ binding site^25^, in Kv7.1 this linker is not conserved.

In summary, combining MD simulations and experiments, we explored the two principal components of coupling in Kv7.1 mediated by protein-protein and protein-lipid interactions. Though, in our simulations, a minimalist truncated model of Kv7.1 was considered, using this, we were able to rationalize the experimental data^9^ and to predict an interaction playing a key role in the protein-protein coupling that was further validated experimentally. Altogether, this brings confidence in the results obtained here: PIP_2_-dependent coupling is especially prominent in this channel due to weakened protein-protein interactions.

While Kv7.1 is functional when it is expressed alone, most of the time, this channel forms complexes with the KCNE auxiliary subunits. For instance, in the heart, Kv7.1 is associated with KCNE1, and this complex produces I_KS_ current, which plays a key role during late phase action potential repolarization. Hence, it is of particular importance to test whether the PIP_2_-dependent mechanism of coupling and the interaction between S4-S5 and S6 are similar in Kv7.1 alone and in its complex with an auxiliary subunit. In order to address this question, further investigations are needed.

In other channels with similar overall architecture (Kv7.2-Kv7.5 and Kv1.2), a relative contribution from the two components of coupling potentially provides a spectrum of responses to voltage and PIP_2_ application as gathered from a survey of their behavior. Whether the VSD/PD coupling is always mediated by the two components in voltage-gated channels requires further thorough investigation. Another important question, concerning the elements involved in the two components specifically, is whether other lipids besides PIP_2_ might contribute to the coupling between the VSD to the PD. We believe, in light of the present study, that a combined modeling and experimental approach would be the best tool to investigate these outstanding issues.

## Methods

### Building Kv7.1 in the activated/open and resting/closed states

The models of the Kv7.1 channel in its activated/open and resting/closed states were built using homology modeling. As templates, we considered respectively the Kv1.2 crystal structure^14^ and the Kv1.2 model obtained from the previous MD simulations^19^. The latter represents the Kv1.2 resting/closed state and is consistent with the models obtained by several other computational groups^20^,^34^. The sequence alignment between Kv1.2 and Kv7.1 performed with ClustalW2^35^ is present in Supplementary Fig. S1. The estimated percentage of identity is 36% for the PD. For the VSD this percentage is lower due to the contribution of the S2–S3 loop, which is drastically different in Kv1.2 and Kv7.1. In order to properly model this part of the channel, we took advantage of the recent NMR data^36^. According to this data, the S2–S3 loop of Kv7.1 adopts a conformation of an α-helix. The alignment of the rest part of the VSD (the S1, S2, S3 and S4 helices and the S1–S2 and S3–S4 loops) was corrected manually considering two criteria: (i) residues of Kv7.1 with important functions should be aligned with residues of similar functions of Kv1.2 (conserved positive residues of S4, conserved negative residues of S2 and S3, conserved phenylalanine on S2); and (ii) insertions or deletions should be pushed to the loop regions. The estimated percentage of identity is 25% for the VSD not considering the S2–S3 loop.

50 different models of each state were prepared using MODELLER^13^. Among them 10 were chosen based on their best values of the DOPE scoring functions. The quality of the models was analyzed using PROCHECK^37^. The structures with the highest number of residues in the core regions of the Ramachandran plot and the lowest number of residues in the disallowed regions were selected for the current study. They contained more than 95% of residues in the core regions and less than 5% of residues in the disallowed regions.

### Preparing the systems for an MD run

For both states (activated/open and resting/closed), the channel was initially embedded in a palmitoyl-oleyl-phosphatidylcholine (POPC) hydrated bilayer. Two systems, with an excess of PIP_2_ and with its low concentration, were considered when studying the activated/open state of Kv7.1. For the first system, the lipid molecules of the inner bilayer leaflet closest to the channel were replaced by PIP_2_ (an excess of PIP_2_: 41 PIP_2_ molecules out of 354 lipid molecules). For the second system, only four PIP_2_ molecules were placed at the intrasubunit binding site preserving the stoichiometry 1 lipid : 1 channel subunit (low concentration of PIP_2_: 4 PIP_2_ molecules out of 378 lipid molecules). For the resting/closed state of Kv7.1, we considered the system with a low concentration of PIP_2_ only (4 PIP_2_ molecules out of 338 lipid molecules). All the systems were hydrated by solution at 150 mM KCl salt concentration. CHARMM22 with the CMAP correction and CHARMM36 force fields were used for the protein and POPC respectively^38^,^39^. To describe PIP_2_, the compatible force field developed by Lupyan *et al.* was considered^40^. Water molecules were represented by the TIP3P model^41^.

### Protocols of MD

The following protocol was used for the system with an excess of PIP_2_ in order to identify a binding site in Kv7.1. First, the entire protein was constrained during 2 ns to ensure the relaxation of the surrounding lipids and the solution. In a second step, for 6 ns MD, only the backbone atoms were constrained with gradually decreasing force constants, enabling the reorganization of the side-chain groups. Finally, all constrains were released and the system was further equilibrated during 100 ns. Additional 40 ns of MD simulations were performed in order to verify their convergence.

For the quantitative analysis of salt bridges in the WT systems a different protocol was applied. The protein backbone was constrained during longer time (100 ns). Based on this trajectory, the time evolution of salt bridges formation was monitored. Several residues of Kv7.1 interacted with the PIP_2_ headgroups temporary, revealing different configurations of the system where corresponding salt bridges were either formed or broken. In total, for the activated/open and resting/closed states we have identified 9 and 8 most frequent configurations respectively. These were considered as starting points for the final equilibration step, including gradual release of the protein backbone and subsequent relaxation of the entire system during 100 ns for each. Hence, for the activated/open and resting/closed states, the entire length of their MD trajectories was 900 and 800 ns respectively.

For the mutants' systems of the activated/open and resting/closed states (K183Q/E, R249Q/E and K358E), only one trajectory of 100 ns was considered. We have applied a similar protocol as described previously for the system with an excess of PIP_2_.

For all the systems, the Root Mean Square Deviations (RMSD) from the initial structure of the channel calculated for the backbone atoms reached equilibrium values after ~50 ns.

### Parameters of MD

The MD simulations were performed using NAMD^42^. Langevin dynamics was applied to keep the temperature (300 K) and the pressure (1 atm) constant. The time-step of the simulations was 2.0 fs. The equations of motion were integrated using a multiple time-step algorithm. Short- and long-range forces were calculated every 1 and 2 time-steps respectively. Long-range electrostatics was calculated using Particle Mesh Ewald (PME). The cutoff distance of short-range electrostatics was taken to be 11 Å. A switching function was used between 8 and 11 Å to smoothly bring the vdW forces and energies to 0 at 11 Å. During the calculations, chemical bonds between hydrogen and heavy atoms were constrained to their equilibrium values. Periodic boundary conditions were applied.

### Analysis of MD trajectories

The analysis was performed for the 50–100 ns time-windows of each trajectory, *i.e.* after the RMSDs from the initial channel structure calculated for the backbone atoms reached a plateau. For the system with an excess of PIP_2_, the analysis was additionally performed for the 100–120 ns and 120–140 ns time-windows in order to verify the convergence of the simulations.

To analyze the salt bridges formation between PIP_2_ and Kv7.1, we measured the minimal distance between the nitrogen atoms of arginine and lysine charged groups and the oxygen atoms of the PIP_2_ phosphates. The salt bridges were assumed formed if the calculated distance was less than 3.2 Å. The probabilities of salt bridge formation were simultaneously estimated for four subunits of the channel as a ratio between the number of frames with a formed salt bridge to its total number. The error bars correspond to a standard deviation (SD) calculated between values obtained from several MD runs of the activated/open and resting/closed states of the WT Kv7.1.

In the per residue decomposition analysis, the nonbonded energies of interactions between each residue of the S4–S5 linker and the S6 helix and vice versa were estimated. The nonbonded energy of interactions between two entire regions, S4–S5 and S6, was calculated also. The following residues were taken into account for both analyses: 247 to 260 of S4–S5 and 323 to 358 of S6. In order to estimate an error bar, the last 50 ns part of the trajectory was divided into 5 consecutive time intervals. For each time interval a mean value of nonbonded energy of the S4–S5/S6 interactions was calculated. The standard deviation between these values was considered as an error bar (SE). In total, for the activated/open and resting/closed states of the WT, 9 and 8 trajectories were taken into account respectively. Only one trajectory for each mutant (K183Q/E, R249Q/E and K358E) was analyzed. In the systems with R249E or K358E, frames with the formed R249E-K358 or R249-K358E salt bridges were not considered for the analyses.

### Mutagenesis

Site-directed mutations were introduced using overlap extension and high-fidelity PCR. DNA sequencing confirmed each mutation. RNA was made by in vitro transcription using the mMessage mMachine T7 polymerase kit (Applied Biosystems).

### Channel Expression

A total of 9.2 ng of channel cRNA was injected, using Nanoject (Drummond), into each of stage V-VI, defolliculated oocytes from *Xenopus laevis*. For expression of CiVSP, 2.3 ng of CiVSP RNA was injected simultaneously. The cells were incubated at 18°C for 4–7 d for robust expression in ND96 solution (in mM: 96 NaCl, 2 KCl, 1.8 CaCl_2_, 1 MgCl_2_, 5 Hepes, 0.3 K_2_EDTA).

### Two-electrode voltage-clamp

Whole-cell currents were recorded from oocytes bathed in ND96 solution using a CA-B amplifier (Dagan) in two-electrode voltage-clamp mode. Microelectrodes were pulled to a resistance of 0.3–3 MΩ when filled with 3 M KCl. Signals were sampled at 1 kHz using the Patchmaster acquisition software (HEKA). The holding potential was set to −80 mV throughout.

### Voltage-clamp fluorometry

For voltage-clamp fluorometry, oocytes were labeled on ice for 45 min in 10 μM Alexa 488 C5 maleimide (Life Technologies) in high-potassium depolarizing solution (in mM: 98 KCl, 1.8 CaCl_2_, 1 MgCl_2_, 5 Hepes, pH 7.6). The cells were washed with ND96 and kept on ice until recording. Fluorescent signals were recorded simultaneously with the whole-cell currents in ND96 solution, using a DLMFS (Leica) upright microscope through a FITC filter cube (Leica). Light from a standard 100-W halogen bulb was focused onto the animal pole of the oocyte and emission from the cube was focused on a P20A photodiode. The current from the photodiode was amplified using an EPC10 patch amplified (HEKA), low-pass filtered at 200 Hz, and sampled at 1 kHz using Patchmaster (HEKA).

All recordings were made in room temperature (20–22°C).

### Data Analysis

The baseline fluorescence was fit with a line during the 2 s at the −80 mV holding potential that preceded each test pulse. This linear baseline approximation was extrapolated to the duration of the pulse and ΔF/F was calculated as (F(t) - F_baseline_(t))/F_baseline_(t), where F(t) is the fluorescent intensity at time t (in arbitrary units) and F_baseline_(t) is the extrapolated baseline value at time t.

The Boltzmann equation was used to fit the fluorescence-voltage relationships: Normalized ΔF(V) = PVa(V) = 1/(1 + exp(-z*F*(V - V_1/2_)/RT), where PVa is the voltage-dependent probability of the voltage sensor assuming the activated state, V is the test voltage (in volts), V_1/2 _is the voltage of half-maximal voltage sensor activation, z is the number of elementary charges translocated across the membrane upon VSD activation, T is the absolute temperature, R is the gas constant (in joules per kelvin per mole), and F is the faraday constant (in coulombs per mole).

## Author Contributions

M.A.K., M.A.Z., J.C. and M.T. designed the project. M.A.K. performed the molecular dynamics simulations. M.A.Z. performed the experiments. M.A.K., M.A.Z., J.C. and M.T. wrote the paper.

## Supplementary Material

Supplementary Information Additional data from simulations and experiments (in PDF format)
