# Supplementary material for: PIP2-dependent coupling is prominent in Kv7.1 due to weakened interactions between S4-S5 and S6
Source: Sci Rep. 2015 Jan 6;5:7474. doi: 10.1038/srep07474 (PMC4284513; doi:10.1038/srep07474)
Supplement: Supplementary Information — Additional data from simulations and experiments (in PDF format) [file srep07474-s1.pdf]

PIP$_2$-dependent coupling is prominent in Kv7.1 due to weakened interactions between S4-S5 and S6

Marina A Kasimova, Mark A Zaydman, Jianmin Cui, Mounir Tarek

Supporting information

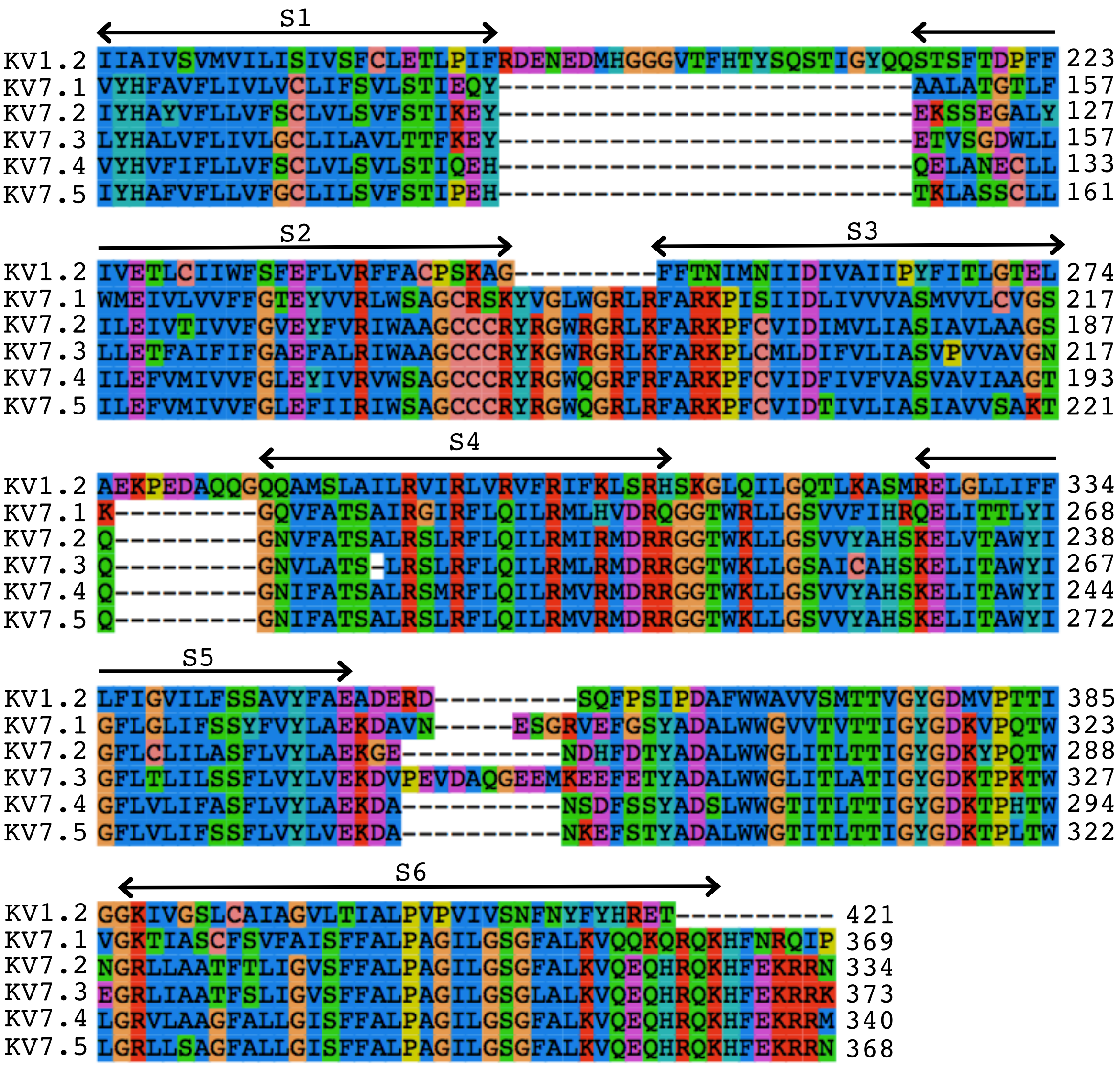

Figure S1 | Multiple sequence alignment between the Kv1.2 channel and the members of the Kv7 family (Kv7.1 to Kv7.5).

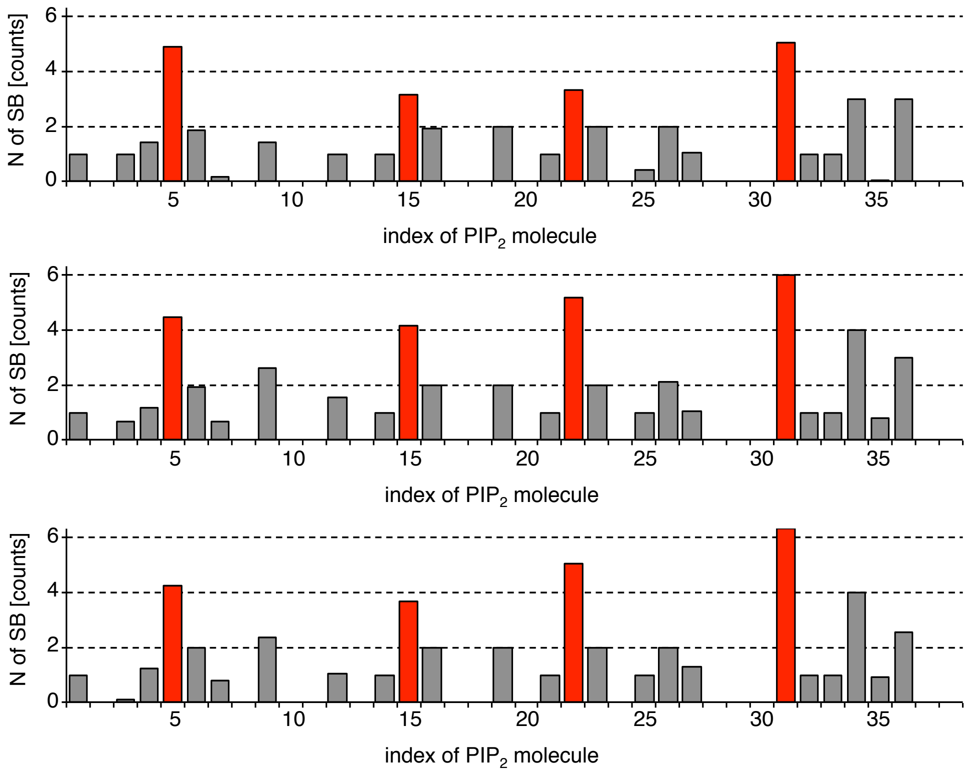

Figure S2 | The average number of salt bridges formed between each PIP_2 molecule (in total 38) and Kv7.1 estimated for the 50-100 (top), 100-120 (middle) and 120-140 (bottom) ns time-windows. The results for the three time-windows are similar, indicating that the simulations have been converged. Red bars correspond to the four PIP_2 molecules bound at the intrasubunit binding site.

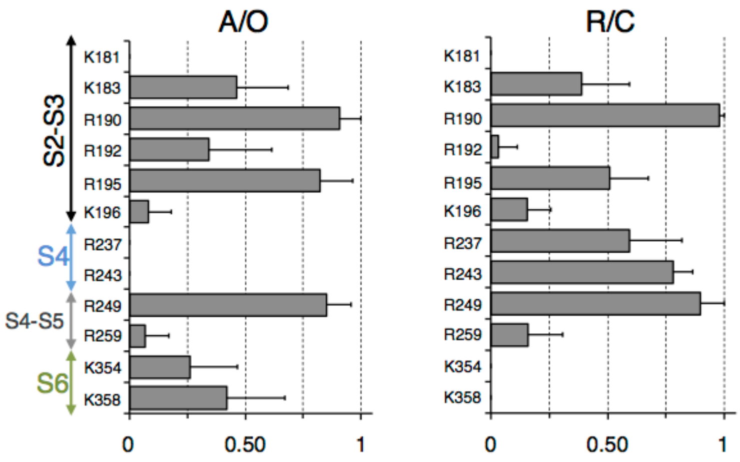

Figure S3 | Two modes of protein-lipid interactions in Kv7.1. Probabilities of salt bridges formation between PIP_2 and several positive residues of Kv7.1 in the activated/open (A/O) and resting/closed (R/C) states.

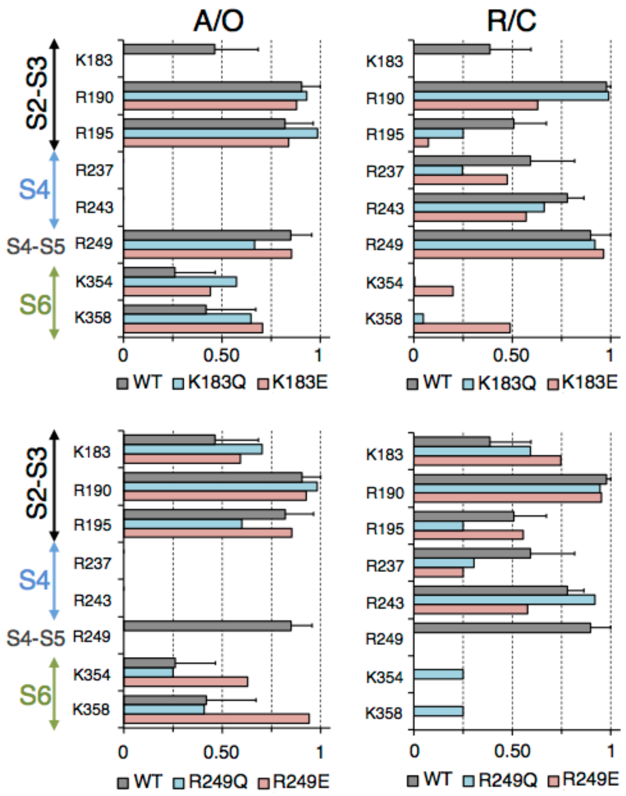

Figure S4 | Probabilities of salt bridges formation between PIP_2 and positive residues of Kv7.1. The results for the WT, the R249 and K183 mutants in their A/O (left panels) and R/C (right panels) states are present.

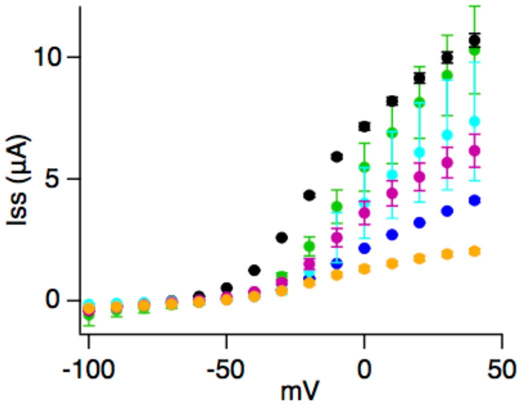

Figure S5 | The I/V curves of the WT (black), K354E (blue), K358E (green), R360E (cyan), K362E (magenta) and R366E (orange) mutants.

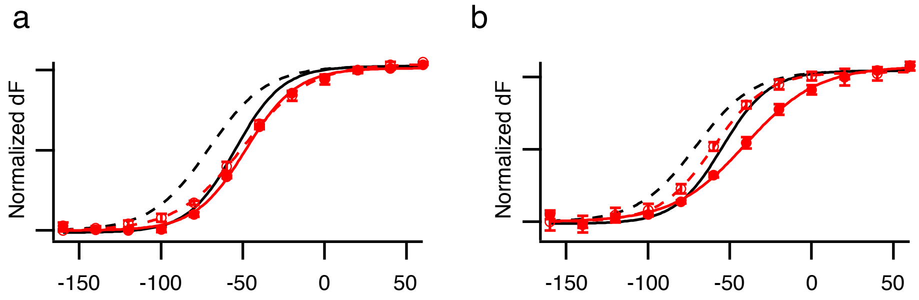

Figure S6 | R555 and R539 residues compose the PIP_2 distal C-terminus site in Kv7.1. (a) The F/V curves of the WT (black solid), L353K (black dashed), R555C (red solid) and R555C/L353K (red dashed). (b) The F/V curves of the WT (black solid), L353K (black dashed), R539W (red solid) and R539W/L353K (red dashed).

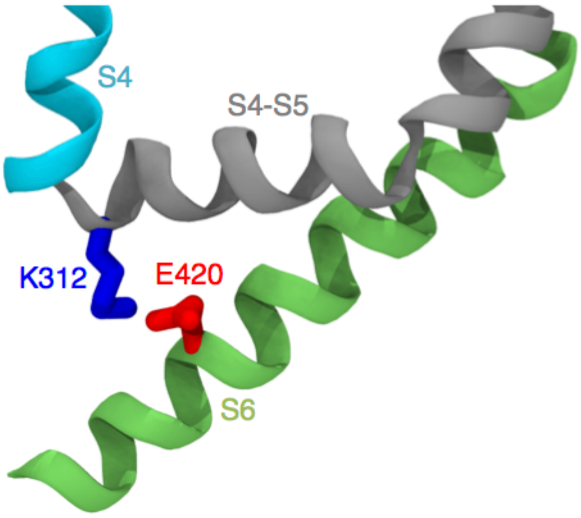

Figure S7 | The salt bridge between K312 (S4-S5) and E420 (S6) in the Kv1.2 channel.

The conformation of the channel was taken from previous simulations[1,2].

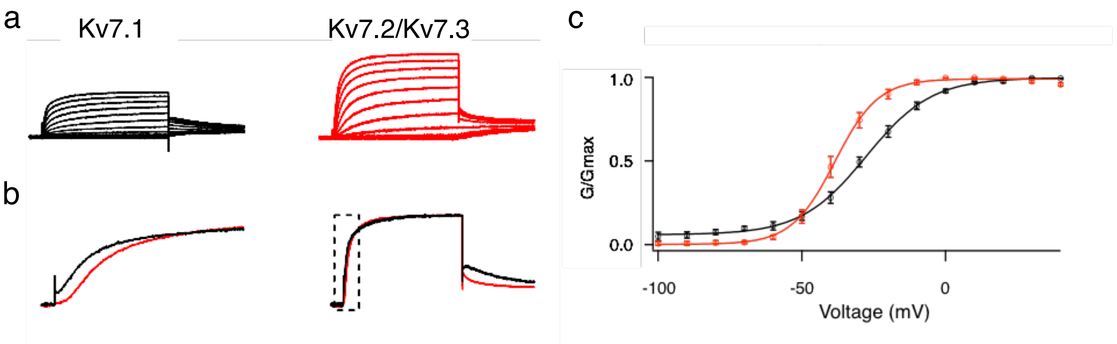

Figure S8 | Comparing properties of the Kv7.1 (black) and Kv7.2/Kv7.3 (red) channels. (a) Currents elicited by a family of depolarizing pulses. (b) Timecourses of current onset; left shows expanded view of dashed boxed region. (c) The G/V curves.

References

1. Delemotte, L., Tarek, M., Klein, M. L., Amaral, C. & Treptow, W. Intermediate states of the Kv1.2 voltage sensor from atomistic molecular dynamics simulations. *Proc. Natl. Acad. Sci. U. S. A.* **108,** 6109–6114 (2011).
2. Kasimova, M. A., Tarek, M., Shaytan, A. K., Shaitan, K. V. & Delemotte, L. Voltage-gated ion channel modulation by lipids: Insights from molecular dynamics simulations. *Biochim. Biophys. Acta* **1838,** 1322–1331 (2014).
